# Preoperative gastric cancer immune prognostic score (GCIPS) as a novel biomarker for predicting survival in gastric cancer patients after radical resection: A retrospective cohort study

**DOI:** 10.1097/MD.0000000000048128

**Published:** 2026-03-20

**Authors:** Xiaosheng Hu, Jinquan Li, Shanzhong Zhang

**Affiliations:** a Department of Gastrointestinal Surgery, Jingdezhen First People’s Hospital, Jingdezhen, Jiangxi, P.R. China.

**Keywords:** gastric cancer, gastric cancer immune prognostic score, GCIPS, prognosis

## Abstract

This study aimed to validate the preoperative gastric cancer immune prognostic score (GCIPS) as a prognostic biomarker in resectable gastric cancer (GC). We retrospectively analyzed 226 GC patients undergoing radical resection. The optimal cutoff value of the GCIPS was determined by receiver operating characteristic curve analysis, and patients were stratified accordingly to assess its prognostic value for recurrence-free survival and overall survival. The GCIPS was calculated using preoperative blood parameters. Using receiver operating characteristic-derived cutoff (2.840), patients were stratified into high- and low-GCIPS groups. The high-GCIPS group showed significantly poorer tumor differentiation (*P* < .001). Kaplan–Meier analysis revealed that high GCIPS was associated with worse 5-year recurrence-free survival (hazard ratio = 2.856, *P* < .001) and overall survival (hazard ratio = 3.222, *P* < .001). Multivariate analysis confirmed GCIPS as an independent predictor for both outcomes after adjusting for tumor-node-metastasis stage and differentiation. The GCIPS is a robust, independent prognostic biomarker derived from routine blood tests, offering a practical tool for risk stratification and guiding individualized management in GC after radical resection.

## 1. Introduction

Gastric cancer (GC) remains one of the most common malignant tumors worldwide, with high incidence and mortality rates, particularly in East Asian regions such as China, where it continues to pose a major public health challenge.^[1]^ Although surgical resection is the primary treatment for GC, especially in early and locally advanced stages, postoperative recurrence and metastasis remain the leading causes of treatment failure and patient mortality.^[2,3]^ Therefore, accurate assessment of postoperative prognosis, particularly in identifying high-risk patients with early recurrence and shorter survival, is critical for developing individualized treatment strategies and optimizing follow-up management.

In recent years, immunotherapy, especially immune checkpoint inhibitors (ICIs), has demonstrated significant efficacy in the treatment of GC. However, its application still faces challenges in patient selection and efficacy prediction.^[4,5]^ Conventional prognostic indicators such as tumor-node-metastasis (TNM) stage, histological type, and tumor markers (e.g., carcinoembryonic antigen [CEA] and carbohydrate antigen 19-9 [CA19-9]) offer certain predictive value but exhibit limitations in highly heterogeneous GC.^[6,7]^ Moreover, while tissue-based biomarkers like PD-L1 combined positive score, microsatellite instability, and tumor mutational burden are crucial for predicting response to immunotherapy, they require specialized testing, can be costly, and are not universally informative.^[8,9]^ In contrast, peripheral blood-based indices like gastric cancer immune prognostic score (GCIPS) provide a noninvasive, systemic assessment of host inflammatory, immune, and coagulation status, offering a complementary and readily accessible prognostic tool.^[10,11]^ Future integrated models combining tissue-based immune scores and blood-based indices may further enhance prognostic precision. Hence, there is growing interest in developing more accurate, convenient, and widely applicable prognostic biomarkers. Given their easy accessibility, low cost, and high reproducibility, hematological parameters have been increasingly utilized in prognostic evaluation of cancers. Inflammatory and nutritional indicators such as the neutrophil-to-lymphocyte ratio (NLR), platelet-to-lymphocyte ratio (PLR), monocytes-to-lymphocyte ratio (MLR), prognostic nutritional index (PNI), and systemic immune-inflammation index (SII), have been confirmed to correlate with prognosis in GC.^[12–15]^ However, most of these parameters were established based on traditional treatment modalities, and their applicability in the context of modern comprehensive therapy remains to be further validated. Moreover, these conventional markers often reflect only isolated aspects of the host’s inflammatory or nutritional status.

Recently, Zuo et al proposed a novel blood-based scoring system (the GCIPS, which was developed based on white blood cell count [WBC], lymphocyte count [LYM], and international normalized ratio [INR]). This score demonstrated excellent prognostic predictive ability in GC patients treated with ICIs.^[16]^ The GCIPS not only reflects systemic inflammatory and immune status but also incorporates multidimensional information related to coagulation function and nutritional condition, indicating its potential as a comprehensive prognostic tool. In contrast to tissue-based biomarkers, peripheral blood-based indices like GCIPS provide a noninvasive, systemic assessment of host inflammatory, immune, and coagulation status, offering a complementary and readily accessible prognostic tool. Future integrated models combining tissue-based immune scores and blood-based indices may further enhance prognostic precision.

However, the prognostic value of GCIPS has not been explored in the surgical context. Given that its components are routinely available preoperatively, we hypothesized that GCIPS could serve as a practical tool for risk stratification in patients undergoing radical resection. Therefore, the primary objective of this study was to validate and extend the applicability of GCIPS by assessing its predictive power for 5-year overall survival (OS) and recurrence-free survival (RFS) in a surgical cohort of GC patients undergoing radical resection. By focusing on radically resected (R0) patients, we aimed to establish a clear and homogeneous cohort, minimizing the confounding prognostic impact of residual tumor burden and allowing for a more accurate assessment of GCIPS’s inherent prognostic power.

## 2. Patients and methods

### 2.1. Study design and objectives

This retrospective cohort study aimed to validate the preoperative GCIPS as a prognostic biomarker in patients with resectable GC. The primary objectives were to assess the association between preoperative GCIPS and 5-year RFS, and 5-year OS. Secondary objectives included evaluating the relationship between GCIPS and clinicopathological features, and comparing its prognostic performance against other inflammatory indices.

### 2.2. Patients

This retrospective study analyzed clinicopathological data and preoperative laboratory hematological parameters (measured within 1 week before surgery) from GC patients who underwent radical resection at Jingdezhen First People’s Hospital, China, between January 2011 and December 2019. A consecutive series of patients meeting the eligibility criteria during this period was initially screened. The inclusion criteria were as follows: histologically confirmed primary GC; no prior neoadjuvant therapy; and curative-intent R0 resection. Patients were excluded based on the following criteria: synchronous or metachronous malignancies; underlying hematological diseases; preoperative infection or immunodeficiency; incomplete medical records; non-radical resection; or receipt of neoadjuvant treatment. Eligible patients were then stratified into high- or low-GCIPS groups based on the optimal cutoff value determined by receiver operating characteristic (ROC) curve analysis (see Section 2.5). All data were retrieved from the hospital’s prospectively maintained database, including detailed surgical reports and electronic medical records from which information on intraoperative blood loss and postoperative complications such as anastomotic leakage was extracted.

### 2.3. Treatment and follow-up

Disease staging was performed according to the 8th edition of the American Joint Committee on Cancer TNM classification.^[17]^ Preexisting comorbidities, including cardiovascular, pulmonary, diabetic, chronic kidney, and chronic liver diseases, were documented. Postoperative anastomotic leakage was specifically defined as leakage occurring within 30 days after surgery. Adjuvant therapy after surgery mainly consisted of chemotherapy, radiotherapy, and related treatment modalities. According to the GC Diagnosis and Treatment Guidelines, the following standardized postoperative follow-up protocol was implemented: for the first 2 years after surgery, patients underwent comprehensive follow-up every 3 months, including medical history collection, physical examination, complete blood count, biochemical tests, and tumor marker assays (such as CEA, CA19-9, and CA125); contrast-enhanced computed tomography scans of the chest, abdomen, and pelvis were performed every 6 months. From the 3rd to the 5th year after surgery, the follow-up interval was extended to every 6 months, with the same examination items. Follow-up was conducted through standardized outpatient visits or structured telephone interviews, and all data were recorded in real time in the hospital database. The follow-up deadline was December 31, 2024, or the date of patient death. For outcome evaluation, RFS, one of the co-primary endpoints, was calculated from the date of surgery to the first recurrence of GC, last follow-up, or death from any cause. OS, the other co-primary endpoint, was defined as the time from surgery to death or the last confirmed follow-up for surviving patients.

### 2.4. Determination of inflammatory markers

All calculations for inflammatory markers can be found in Table 1, with the GCIPS calculated as follows: white blood cells (10^9^/L) × 0.071 ‐ lymphocytes (10^9^/L) × 0.375 + international normalized ratio × 2.986.^[12]^

**Table 1:** Full names, abbreviations, calculation formulas, and optimal cutoff values of the markers.

Abbreviation of markers	Full name of the marker	Calculation formula	Optimal cutoff value
GCIPS	Gastric cancer immune prognostic score	White blood cells (10^9^/L) × 0.071 ‐ lymphocytes (10^9^/L) × 0.375 + international normalized ratio × 2.986	2.837
NLR	Neutrophil-to-lymphocyte ratio	Neutrophils (10^9^/L)/lymphocytes (10^9^/L)	2.522
PLR	Platelet-to-lymphocyte ratio	Platelets (10^9^/L)/lymphocytes (10^9^/L)	134.963
MLR	Monocytes-to-lymphocyte ratio	Monocytes (10^9^/L)/lymphocytes (10^9^/L)	0.266
PNI	Prognostic nutritional index	Albumin (g/L) + 5 × lymphocytes (10^9^/L)	46.425
SII	Systemic immune-inflammation index	Platelets (10^9^/L) × neutrophils (10^9^/L)/lymphocytes (10^9^/L)	552.385

GCIPS = gastric cancer immune prognostic score, MLR = monocyte-to-lymphocyte ratio, NLR = neutrophil-to-lymphocyte ratio, PLR = platelet-to-lymphocyte ratio, PNI = prognostic nutritional index, SII = systemic immune-inflammation index.

### 2.5. Statistical analysis

All statistical analyses were conducted with R software (version 4.3.3; R Foundation for Statistical Computing, Vienna, Austria). Key packages included pROC (v1.18.5) for ROC curve analysis, wherein the optimal cutoff for GCIPS was determined by maximizing the Youden index based on the area under the curve for predicting 5-year OS; survival (v3.6.4) and survminer (v0.4.9) for survival modeling; and rstatix (v0.7.2) for general statistical tests. According to this optimal cutoff, patients were categorized into high- and low-GCIPS groups for all subsequent analyses. Normally distributed continuous variables are summarized as mean ± standard deviation and compared via independent samples *t* tests; non-normal continuous variables are reported as median (interquartile range, Q1–Q3) and analyzed using Wilcoxon rank-sum tests. Categorical variables are presented as frequencies (percentages) and compared with χ^2^ tests or Fisher exact tests, as appropriate. All *P*-values were two-tailed, with significance set at *P* < .05. To address potential confounding, particularly by adjuvant therapy, both univariate and multivariate Cox proportional hazards models were pre‑specified. Variables with *P* < .05 in the univariate analysis, including adjuvant therapy, were included in the multivariate Cox model to compute hazard ratios (HRs) and 95% confidence intervals (CIs) for RFS and OS and to identify independent prognostic factors. Survival curves were generated with the Kaplan–Meier method, and group differences were evaluated with log-rank tests.

### 2.6. Ethical approval

The present study was conducted in accordance with the Declaration of Helsinki and approved by the Research Ethics Committee at Jingdezhen First People’s Hospital (approval no. jdzyy202537). The requirement for informed consent was waived due to the retrospective nature of the study and data anonymization.

## 3. Results

### 3.1. Patient characteristics

**Figure 1.**
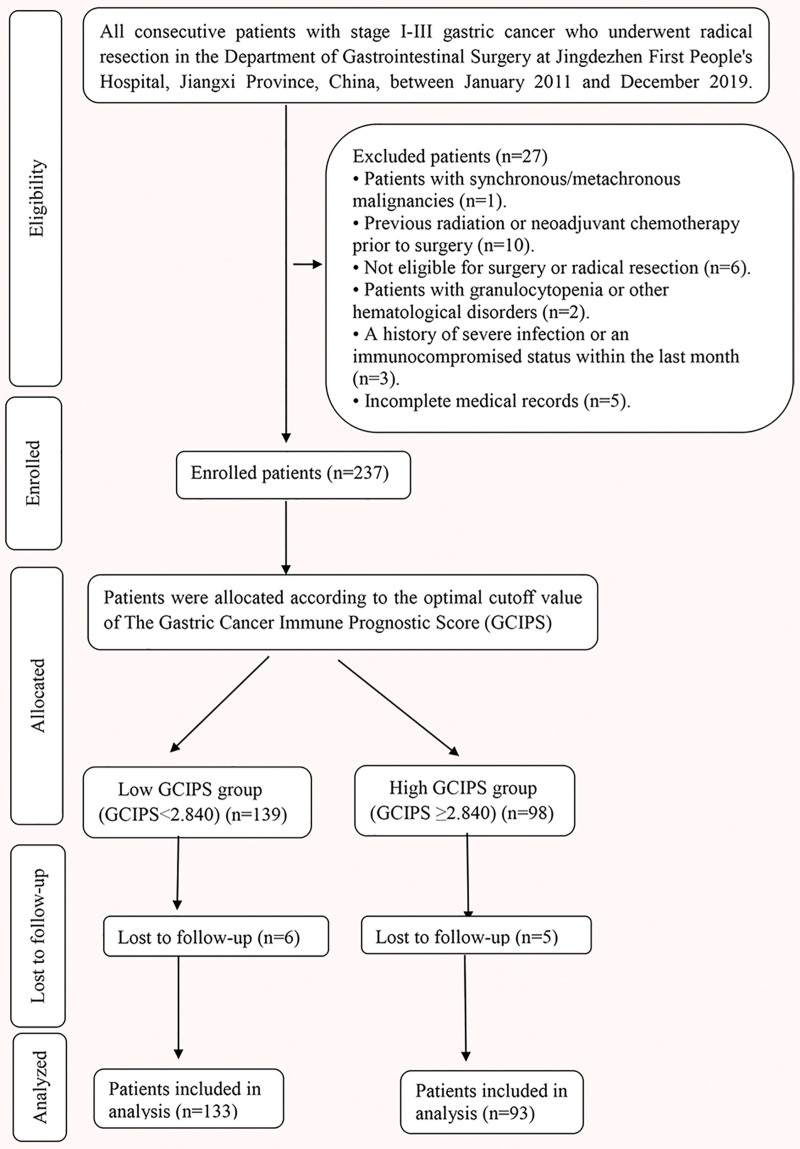
Screening flowchart for gastric cancer patients undergoing radical resection.

**Figure 2.**
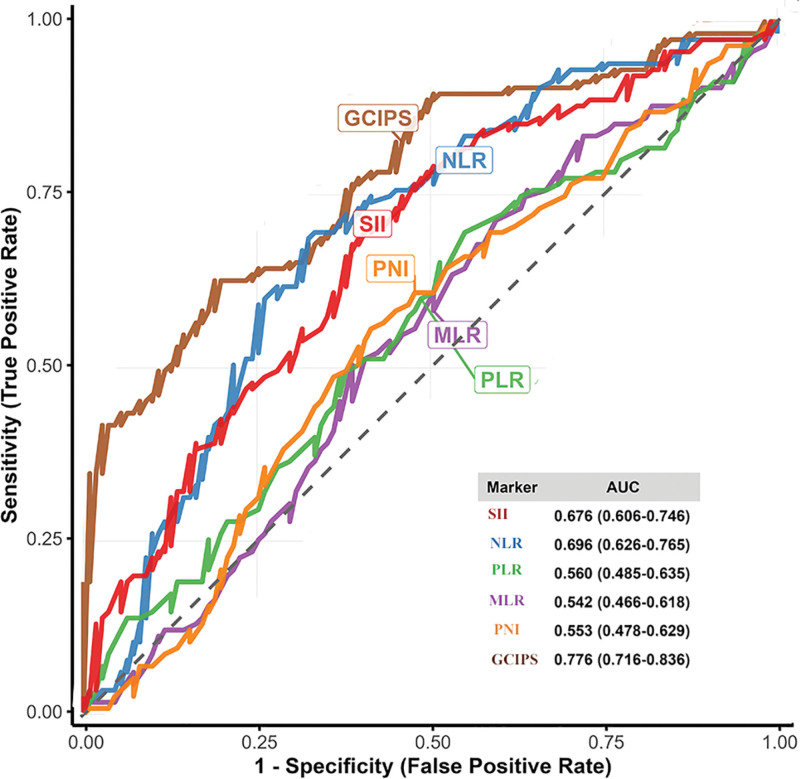
Receiver operating characteristic curve for the inflammatory makers. AUC = area under the curve.

A total of 237 patients with GC who underwent radical resection between January 2011 and December 2019 were initially enrolled in this study (Fig. 1). After applying the exclusion criteria (n = 27) and accounting for loss to follow-up (n = 11; 6 from the low-GCIPS and 5 from the high-GCIPS group), a final total of 226 patients were included in the analysis (low-GCIPS, n = 133; high-GCIPS, n = 93). Using the optimal cutoff value of 2.840 for GCIPS, patients were stratified into low-GCIPS (n = 133) and high-GCIPS (n = 93) groups. ROC curve analysis (Fig. 2) was performed to evaluate and compare the predictive performance of multiple inflammatory, immune, and nutritional markers, including GCIPS, SII, PNI, MLR, NLR, and PLR. Among all markers, GCIPS demonstrated the highest discriminative ability, with an area under the curve of 0.776 (95% CI: 0.716–0.836). This indicates that GCIPS had the best predictive performance for clinical outcomes among the biomarkers compared. Clinicopathological characteristics were well balanced between the 2 groups for most baseline variables (Table 2). No significant differences were observed in age (*P* = .699), gender (*P* = .252), tumor location (*P* = .884), surgical approach (*P* = .719), anastomotic method (*P* = .734), tumor size (*P* = .824), intraoperative blood loss (*P* = .672), comorbidities (*P* = .416), anemia (*P* = .794), pyloric stenosis (*P* = .792), transfusion requirement (*P* = .872), or anastomotic leakage (*P* = .487). Similarly, no significant intergroup differences were detected in T stage (*P* = .450), N stage (*P* = .209), TNM stage (*P* = .553), rates of postoperative adjuvant therapy (*P* = .967), or preoperative tumor marker levels including CEA > 5 ng/mL (*P* = .323), CA19-9 > 30 U/mL (*P* = .532), and CA-125 > 25 U/mL (*P* = .990). This general balance in baseline characteristics reduces potential confounding and strengthens the validity of the subsequent survival comparisons between the GCIPS groups.

**Table 2:** Baseline clinicopathological characteristics of gastric cancer patients stratified by GCIPS.

Variable	Total (n = 226)	GCIPS		*P*
		Low (n = 133)	High (n = 93)	
Age (yr) (mean ± SD)	61.10 ± 10.83	61.33 ± 10.53	60.76 ± 11.28	.699
Gender, n (%)				.252
Male	167 (73.89)	102 (76.69)	65 (69.89)	
Female	59 (26.11)	31 (23.31)	28 (30.11)	
Location, n (%)				.884
Upper	24 (10.62)	13 (9.77)	11 (11.83)	
Middle	67 (29.65)	40 (30.08)	27 (29.03)	
Lower	135 (59.73)	80 (60.15)	55 (59.14)	
Surgery, n (%)				.719
Open	180 (79.65)	107 (80.45)	73 (78.49)	
Laparoscopic	46 (20.35)	26 (19.55)	20 (21.51)	
Anastomotic method, n (%)				.734
Billroth I	59 (26.10)	33 (24.81)	26 (27.96)	
Billroth II	136 (60.18)	80 (60.15)	56 (60.22)	
Roux-en-Y	31 (13.72)	20 (15.04)	11 (11.82)	
Tumor size (cm) [M (Q_1_, Q_3_)]	[3.00 (2.50, 5.00)]	[3.00 (2.50, 5.00)]	[3.00 (2.60, 4.00)]	.824
Blood loss (mL) [M (Q_1_, Q_3_)]	[100.0 (100.0, 150.0)]	[100.0 (100.0, 200.0)]	[100.0 (100.0, 150.0)]	.672
Comorbidities, n (%)				.416
No	189 (83.63)	109 (81.95)	80 (86.02)	
Yes	37 (16.37)	24 (18.05)	13 (13.98)	
Anemia, n (%)				.794
No	196 (86.73)	116 (87.22)	80 (86.02)	
Yes	30 (13.27)	17 (12.78)	13 (13.98)	
Pyloric stenosis, n (%)				.792
No	214 (94.69)	125 (93.98)	89 (95.70)	
Yes	12 (5.31)	8 (6.02)	4 (4.30)	
Transfusion, n (%)				.872
No	193 (85.40)	114 (85.71)	79 (84.95)	
Yes	33 (14.60)	19 (14.29)	14 (15.05)	
Anastomotic leakage, n (%)				.487
No	212 (93.81)	126 (94.74)	86 (92.47)	
Yes	14 (6.19)	7 (5.26)	7 (7.53)	
Tumor differentiation, n (%)				**<.001**
Well	29 (12.83)	25 (18.80)	4 (4.30)	
Moderate	164 (72.57)	98 (73.68)	66 (70.97)	
Poor	33 (14.60)	10 (7.52)	23 (24.73)	
T stage, n (%)				.450
1	14 (6.19)	8 (6.02)	6 (6.45)	
2	47 (20.80)	24 (18.05)	23 (24.73)	
3	32 (14.16)	17 (12.78)	15 (16.13)	
4	133 (58.85)	84 (63.16)	49 (52.69)	
N stage, n (%)				.209
0	81 (35.84)	53 (39.85)	28 (30.11)	
1	90 (39.82)	54 (40.60)	36 (38.71)	
2	36 (15.93)	17 (12.78)	19 (20.43)	
3	19 (8.41)	9 (6.77)	10 (10.75)	
TNM stage, n (%)				.553
I	40 (17.70)	26 (19.55)	14 (15.05)	
II	75 (33.19)	41 (30.83)	34 (36.56)	
III	111 (49.12)	66 (49.62)	45 (48.39)	
P-adjuvant therapy, n (%)				.967
No	58 (25.66)	34 (25.56)	24 (25.81)	
Yes	168 (74.34)	99 (74.44)	69 (74.19)	
CEA > 5 ng/mL, n (%)				.323
No	182 (80.53)	110 (82.71)	72 (77.42)	
Yes	44 (19.47)	23 (17.29)	21 (22.58)	
CA199 > 30 U/mL, n (%)				.532
No	198 (87.61)	115 (86.47)	83 (89.25)	
Yes	28 (12.39)	18 (13.53)	10 (10.75)	
CA125 > 25 U/mL, n (%)				.990
No	216 (95.58)	127 (95.49)	89 (95.70)	
Yes	10 (4.42)	6 (4.51)	4 (4.30)	
GCIPS, M (Q_1_, Q_3_)	[2.72 (2.46, 3.06)]	[2.51 (2.25, 2.66)]	[3.12 (2.98, 3.34)]	**<.001**

Data are presented as mean ± SD, n (%), or median (IQR). *P*-values were derived from independent samples *t* test (for age), Chi-squared test or Fisher exact test (for categorical variables), and Mann–Whitney *U* test (for non-normally distributed continuous variables: tumor size, blood loss, GCIPS value). A *P*-value < .05 was considered statistically significant and highlighted in bold.

CA-125 = carbohydrate antigen 125, CA19-9 = carbohydrate antigen 19-9, CEA = carcinoembryonic antigen, GCIPS = gastric cancer immune prognostic score, IQR = interquartile range, P-adjuvant therapy = postoperative adjuvant therapy, SD = standard deviation, TNM = tumor-node-metastasis.

However, significant differences were identified in tumor differentiation (*P* < .001). The high-GCIPS group had a substantially higher proportion of poorly differentiated (poor) tumors (24.73% vs 7.52%; HR = 3.12, 95% CI: 1.45–6.72, *P* < .001) and a lower proportion of well-differentiated (well) tumors (4.30% vs 18.80%) compared to the low-GCIPS group. As expected, the median GCIPS values significantly differed between the 2 groups [low-GCIPS: 2.51, 95% CI: 2.25–2.66 vs high-GCIPS: 3.12, 95% CI: 2.98–3.34; *P* < .001].

In summary, while most baseline and clinical characteristics were comparable between the groups, a high GCIPS was significantly associated with a more aggressive pathological pattern, specifically poorer tumor differentiation. This specific association suggests that the systemic inflammatory, immune, and coagulation milieu reflected by a high GCIPS may be linked to underlying biological aggressiveness.

#### 3.1.1. Prognostic significance of GCIPS in GC survival

**Figure 3.**
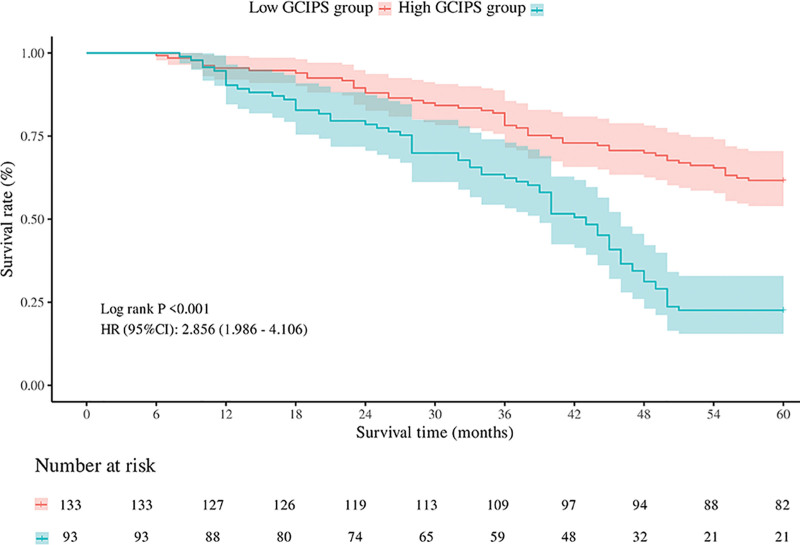
RFS in patients with gastric cancer in the GCIPS-high and GCIPS-low groups. GCIPS = gastric cancer immune prognostic score; RFS = recurrence-free survival.

**Figure 4.**
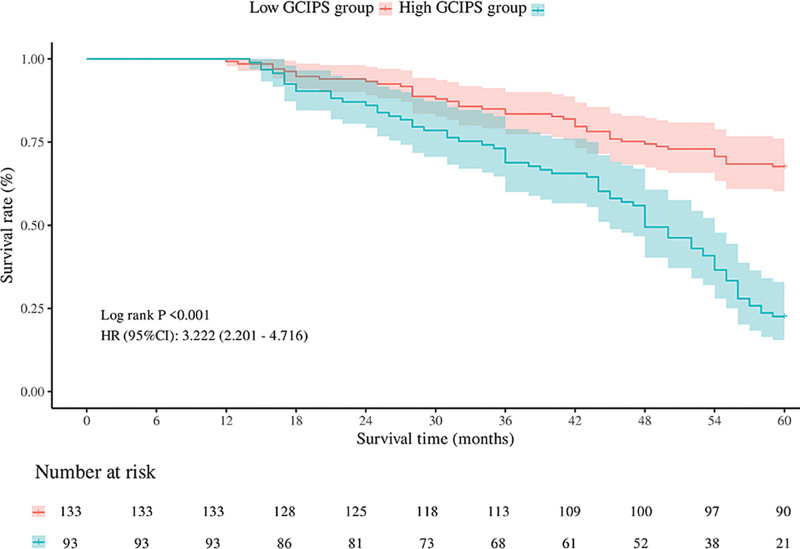
OS in patients with gastric cancer in the GCIPS-high and GCIPS-low groups. GCIPS = gastric cancer immune prognostic score; OS = overall survival.

Kaplan–Meier survival analysis was performed to evaluate the prognostic significance of GCIPS in GC patients. As shown in Figure 3, patients in the high-GCIPS group had significantly worse RFS compared to those in the low-GCIPS group (log-rank *P* < .001). The HR for recurrence in the high-GCIPS group was 2.856 (95% CI: 1.986–4.106). Similarly, as illustrated in Figure 4, OS was also significantly reduced in the high-GCIPS group (log-rank *P* < .001), with an HR of 3.222 (95% CI: 2.201–4.716). The number at risk tables confirm a consistent decline in survival probability over time in both groups, with more pronounced event occurrences in the high-GCIPS cohort. These results strongly indicate that elevated GCIPS is associated with unfavorable survival outcomes in GC.

#### 3.1.2. COX regression analysis of 5-year RFS in patients with GC

Univariate and multivariate Cox regression analyses were performed to identify prognostic factors for RFS in GC patients (Table 3). Univariate analysis revealed that several factors were significantly associated with worse RFS, including poor tumor differentiation (poor vs well differentiated: HR = 7.88, 95% CI: 3.58–17.31, *P* < .001), advanced TNM stage (III vs I: HR = 8.71, 95% CI: 4.03–18.86, *P* < .001), elevated CEA level (>5 vs ≤5 ng/mL: HR = 1.76, 95% CI: 1.18–2.63, *P* = .005), high GCIPS (high vs low: HR = 2.86, 95% CI: 1.99–4.11, *P* < .001), as well as transfusion (yes vs no: HR = 1.65, 95% CI: 1.04–2.62, *P* = .033), N stage (N0/N1 vs N2/N3: HR = 0.75, 95% CI: 0.75 (0.54–0.97), *P* = .042), and adjuvant therapy (yes vs no: HR = 1.80, 95% CI: 1.14–2.83, *P* = .011).

**Table 3:** Univariate and multivariate analysis of factors associated with recurrence-free survival in gastric cancer patients.

Variables	Univariate analysis		Multivariate analysis	
	HR (95 CI)	*P*-value	HR (95 CI)	*P*-value
Gender, female versus male	0.92 (0.62–1.37)	.691		
Age, ≥60 versus <60 years	1.08 (0.75–1.56)	.663		
Location, middle and lower versus upper	0.83 (0.60–1.50)	.263		
Surgery, laparoscopic vs open	0.77 (0.46–1.30)	.334		
Tumor size, ≥3.5 versus <3.5 cm	1.14 (0.80–1.62)	.481		
Blood loss, ≥100 versus <100 mL	1.10 (0.76–1.60)	.618		
Comorbidities, yes vs no	1.06 (0.66–1.69)	.822		
Anemia yes, yes vs no	1.54 (0.96–2.49)	.076		
Pyloric stenosis, yes vs no	0.99 (0.46–2.13)	.986		
Transfusion, yes vs no	1.65 (1.04–2.62)	**.033**	1.11 (0.49–2.51)	.796
Anastomotic method, Billroth I/II vs Roux-en-Y	0.96 (0.87–1.07)	.481		
Anastomotic leakage, yes vs no	1.58 (0.83–3.02)	.160		
Tumor differentiation				
Well	1.00 (Reference)		1.00 (Reference)	
Moderate	2.20 (1.06–4.54)	**.033**	2.54 (1.12–5.75)	**.025**
Poor	7.88 (3.58–17.31)	**<.001**	4.80 (1.86–12.41)	**.001**
T stage, T1/T2 versus T3/T4	0.66 (0.42–1.01)	.056		
N stage, N0/N1 versus N2/N3	0.75 (0.54–0.97)	**.042**	0.87 (0.74–1.02)	.089
TNM stage				
I	1.00 (Reference)		1.00 (Reference)	
II	2.25 (0.98–5.17)	.056	2.24 (0.57–8.82)	.249
III	8.71 (4.03–18.86)	**<.001**	14.95 (1.92–116.64)	**.010**
P-adjuvant therapy, yes vs no	1.80 (1.14–2.83)	**.011**	1.30 (0.74–2.28)	.359
CEA, >5 versus ≤5 ng/mL	1.76 (1.18–2.63)	**.005**	0.65 (0.39–1.11)	.113
CA125, >25 versus ≤25 U/mL	1.44 (0.67–3.10)	.345		
CA199, >30 vs ≤30 U/mL	1.31 (0.78–2.19)	.303		
GCIPS, high versus low	2.86 (1.99–4.11)	**<.001**	2.43 (1.49–3.97)	**<.001**

Statistically significant *P*-values (<.05) are highlighted in bold. Multivariate analysis was adjusted for variables with *P* < .05 in the univariate analysis.

CA-125 = carbohydrate antigen 125, CA19-9 = carbohydrate antigen 19-9, CEA = carcinoembryonic antigen, CI = confidence interval, GCIPS = gastric cancer immune prognostic score, HR = hazard ratio, P-adjuvant therapy = postoperative adjuvant therapy, TNM = tumor-node-metastasis.

Multivariate analysis confirmed that high GCIPS remained an independent predictor of poor RFS (HR = 2.43, 95% CI: 1.49–3.97, *P* < .001), along with poor tumor differentiation (moderate vs well: HR = 2.54, 95% CI: 1.12–5.75, *P* = .025; poor vs well: HR = 4.80, 95% CI: 1.86–12.41, *P* = .001) and advanced TNM stage (III vs I: HR = 14.95, 95% CI: 1.92–116.64, *P* = .010). However, factors such as transfusion, N stage, adjuvant therapy, and elevated CEA, which were significant in univariate analysis, lost statistical significance in the multivariate model. This suggests that the prognostic impact of these factors on recurrence risk may be largely mediated or confounded by their association with the systemic inflammatory-immune-coagulation state captured by GCIPS, inherent tumor aggressiveness (differentiation), and anatomical disease extent (TNM stage). *COX regression analysis of 5-year OS in patients with GC.* Univariate and multivariate Cox regression analyses were performed to identify prognostic factors for OS in GC patients (Table 4). Univariate Cox regression analysis identified several factors significantly associated with worse OS in GC patients, including poor tumor differentiation (poor vs well differentiated: HR = 9.78, 95% CI: 4.05–23.64, *P* < .001), advanced TNM stage (stage III vs I: HR = 7.45, 95% CI: 3.44–16.15, *P* < .001), elevated CEA level (>5 vs ≤5 ng/mL: HR = 1.67, 95% CI: 1.10–2.53, *P* = .016), high GCIPS (high vs low: HR = 3.22, 95% CI: 2.20–4.72, *P* < .001), and receipt of adjuvant therapy (yes vs no: HR = 1.71, 95% CI: 1.07–2.72, *P* = .024).

**Table 4:** Univariate and multivariate analysis of factors associated with overall survival in gastric cancer patients.

Variables	Univariate analysis			Multivariate analysis		
	HR (95 CI)	*P*-value		HR (95 CI)	*P*-value	
Gender, female versus male	0.88 (0.59–1.32)	.538				
Age, ≥60 versus <60 years	1.09 (0.75–1.59)	.658				
Location, middle and lower versus upper	0.98 (0.71–1.35)	.879				
Surgery, laparoscopic versus open	0.79 (0.48–1.30)	.351				
Tumor size, ≥3.5 versus <3.5 cm	1.11 (0.77–1.61)	.570				
Blood loss, ≥100 versus <100 mL	1.05 (0.71–1.55)	.808				
Comorbidities, yes vs no	0.88 (0.52–1.47)	.618				
Anemia yes, yes vs no	1.44 (0.87–2.38)	.157				
Pyloric stenosis, yes vs no	0.93 (0.41–2.12)	.870				
Transfusion, yes vs no	1.60 (0.99–2.56)	.053				
Anastomotic method, Billroth I/II vs Roux-en-Y	0.86 (0.75–1.95)	.120				
Anastomotic leakage, yes vs no	1.53 (0.77–3.02)	.223				
Tumor differentiation						
Well	1.00 (Reference)			1.00 (Reference)		
Moderate	2.72 (1.18–6.23)	.018		3.18 (1.25–8.05)	**.015**	
Poor	9.78 (4.05–23.64)	**<.001**		6.34 (2.21–18.20)	**<.001**	
T stage, T1/T2 vs T3/T4	0.72 (0.55–1.23)	.075				
N stage, N0/N1 vs N2/N3	0.78 (0.56–1.12)	**.035**	0.89 (0.66–1.34)			.158
TNM stage						
I	1.00 (Reference)			1.00 (Reference)		
II	2.11 (0.91–4.85)	.080	2.23 (0.53–9.28)			.272
III	7.45 (3.44–16.15)	**<.001**	15.45 (1.74–137.42)			**.014**
P-adjuvanttherapy, yes vs no	1.71 (1.07–2.72)	**.024**	1.32 (0.73–2.38)			.361
CEA, >5 versus ≤5 ng/mL	1.67 (1.10–2.53)	**.016**	0.65 (0.38–1.11)			.116
CA125, >25 versus ≤25 U/mL	1.57 (0.73–3.38)					
CA199, >30 versus ≤30 U/mL	1.37 (0.82–2.30)	.226				
GCIPS, high vs low	3.22 (2.20–4.72)	**<.001**	2.43 (1.49–3.97)			**.001**

Statistically significant *P*-values (<.05) are highlighted in bold. Multivariate analysis was adjusted for variables with *P* < .05 in the univariate analysis.

CA-125 = carbohydrate antigen 125, CA19-9 = carbohydrate antigen 19-9, CEA = carcinoembryonic antigen, CI = confidence interval, GCIPS = gastric cancer immune prognostic score, HR = hazard ratio, P-adjuvanttherapy = postoperative adjuvant therapy, TNM = tumor-node-metastasis.

Multivariate analysis demonstrated that GCIPS remained a strong independent prognostic factor for OS (HR = 2.43, 95% CI: 1.49–3.97, *P* = .001). Additionally, tumor differentiation (moderate vs well: HR = 3.18, 95% CI: 1.25–8.05, *P* = .015; poor vs well: HR = 6.34, 95% CI: 2.21–18.20, *P* < .001) and advanced TNM stage (III vs I: HR = 15.45, 95% CI: 1.74–137.42, *P* = .014) retained their independent prognostic significance. Notably, elevated CEA level and adjuvant therapy, which showed significance in univariate analysis, were not independent predictors in the multivariate model. This reinforces the robustness of preoperative GCIPS as a prognostic indicator that provides information beyond that contained in traditional treatment-related decisions (like adjuvant therapy) or a single tumor marker, reflecting a powerful host systemic status.

## 4. Discussion

The present study demonstrates that the preoperative GCIPS serves as a robust and independent prognostic biomarker for predicting both RFS and OS in patients with GC following radical resection. Our results indicate that a preoperative GCIPS value ≥2.840 is significantly associated with aggressive tumor biology, including poorer histological differentiation and unfavorable survival outcomes. Notably, our findings extend the applicability of the GCIPS, originally developed and validated by Zuo et al in a cohort of patients receiving ICIs,^[16]^ to the context of surgically resected GC. The balance in most baseline clinicopathological variables between the low- and high-GCIPS groups (Table 2) strengthens our study by reducing potential confounding and allowing a clearer attribution of survival differences to the GCIPS itself. The one striking exception was tumor differentiation, which was significantly worse in the high-GCIPS group. This specific association is biologically plausible. A high GCIPS, indicative of systemic inflammation (elevated WBC), relative immune suppression (low LYM), and a pro-thrombotic state (elevated INR), fosters a tumor-promoting microenvironment. An elevated white blood cell count contributes to a milieu rich in proinflammatory cytokines (e.g., IL-6, TNF-α) and myeloid-derived suppressor cells, which can induce genomic instability and stimulate angiogenesis. Lymphopenia reflects impaired immune surveillance and failure of cancer immunoediting, weakening cytotoxic antitumor responses. An abnormal INR promotes tumor growth and metastasis through coagulation protease-activated signaling pathways. Collectively, this triad (chronic inflammation, immune suppression, and activated coagulation) forms a interconnected network that drives tumor progression, epithelial–mesenchymal transition, and cellular dedifferentiation, thereby contributing directly to a more aggressive, poorly differentiated tumor phenotype.^[18–21]^

Accumulating evidence indicates that systemic inflammation and immune dysfunction play pivotal roles in cancer progression and metastasis,^[22]^ which is reflected by inflammatory and nutritional indicators such as the NLR, PLR, MLR, PNI, and SII.^[12–14]^ However, most of these conventional markers reflect only isolated aspects of the host’s immune or nutritional status. In contrast, the innovative significance of the GCIPS lies in its integration of WBC count, LYM count, and INR, thereby providing a more comprehensive multidimensional reflection of the host’s inflammatory status, immune competence, and coagulation function.^[16]^ From a mechanistic perspective, each component of GCIPS carries significant biological implications: an elevated white blood cell count not only indicates a systemic inflammatory response (associated with the release of proinflammatory cytokines [e.g., IL-6, TNF-α] and expansion of myeloid-derived suppressor cells^[23,24]^) but also contributes to a tumor-promoting milieu by inducing genomic instability and stimulating angiogenesis.^[25,26]^ Lymphopenia reflects immune exhaustion and failure of cancer immunoediting,^[27,28]^ whereas intact lymphocyte function (particularly that of cytotoxic T cells and NK cells) is essential for antitumor immunity.^[29]^ An abnormal INR not only signals an increased risk of thrombosis but also promotes tumor growth, angiogenesis, and premetastatic niche formation through tissue factor-mediated activation of the coagulation cascade in the tumor microenvironment and protease-activated receptor signaling pathways activated by coagulation proteases (such as factor *Xa* and thrombin).^[21,30,31]^ Importantly, these 3 pathways (inflammation, immunity, and coagulation) do not act in isolation. Instead, they form a closely interconnected network wherein inflammatory cytokines modulate both coagulation activity and immune cell function.^[32]^ This tripartite interaction constitutes a complex biological network of tumor–host interactions.^[33]^ By integrating these 3 key pathways, the GCIPS comprehensively captures the global state of this network, which fully accounts for its exceptional prognostic value and clinical superiority.^[21,33,34]^

The findings of the present study are highly consistent with those reported by Zuo et al^[16]^ and further extend the applicability of the GCIPS from patients with advanced disease ineligible for ICI therapy to GC patients undergoing radical resection. Notably, in the multivariate analysis of this study, GCIPS remained an independent predictor of prognosis (RFS: HR = 2.43; OS: HR = 2.43) even after adjusting for traditional strong prognostic factors such as TNM stage and tumor differentiation. This result strongly suggests that the systemic inflammatory, immune, and coagulation status captured by GCIPS provides unique biological insights beyond traditional anatomic staging systems, aiding in the identification of patient subgroups with vastly different actual prognoses who are classified into the same stage by conventional criteria.^[35,36]^ It is worth noting that factors like adjuvant therapy, which was significant in univariate analysis, lost its independent prognostic value in the multivariate model. This suggests that the decision to administer adjuvant therapy may often be based on clinical assessments that already correlate with the underlying high-risk biology that GCIPS objectively quantifies.

The clinical utility of GCIPS is also reflected in the simplicity of its components. All indicators (WBC, LYM, and INR) are derived from routine preoperative blood tests, requiring no additional costs and are easily standardized across healthcare institutions at various levels.^[37]^ This makes GCIPS a highly cost-effective prognostic stratification tool, particularly suitable for regions with limited medical resources. Obtaining the GCIPS score preoperatively can provide crucial reference for clinicians in perioperative decision-making. For instance, for patients with a high GCIPS score, even those at a relatively early stage, more aggressive adjuvant therapy strategies and closer follow-up monitoring could be considered to facilitate early detection and intervention of recurrence and metastasis.^[38]^ While our study focused on radically resected patients to establish a clear prognostic signal, future research should explore the utility of GCIPS in patients undergoing partial or non-radical resections, where the systemic host environment might play an even more critical role in outcome.

Certainly, this study has several limitations. First, as a single-center retrospective study, it carries inherent selection bias. Second, the study population was sourced from a single region in China, and the generalizability of its conclusions to external populations (different ethnicities, regions) requires further validation. Third, the optimal cutoff value for GCIPS (2.840) was derived from ROC analysis of this study’s data; its universality and stability need external validation in prospective, multicenter, large-sample cohorts. Finally, molecular subtypes of tumors (such as EBV status, microsatellite instability status, and HER2 expression status) have been confirmed to be closely related to the prognosis and treatment response of GC.^[3,39]^ However, this study could not obtain and analyze these data. Future research should explore the combined application value of GCIPS and molecular subtyping to build more accurate prognostic prediction models.

## 5. Conclusion

In summary, this study confirms that the preoperative GCIPS is a robust and independent predictive factor for survival outcomes in patients with GC undergoing radical resection. By integrating the 3 biological pathways of systemic inflammatory response, immune status, and coagulation function, it provides a comprehensive prognostic assessment tool that surpasses traditional clinicopathological indicators. GCIPS is simple to calculate, low-cost, and easy to implement clinically, showing promise as a novel and practical biomarker for the individualized assessment of postoperative risk and for guiding adjuvant therapy and follow-up strategies in GC patients. Future prospective multicenter studies are warranted to validate its cutoff value and explore its potential for combined application with tumor molecular characteristics.

## Author contributions

**Conceptualization:** Xiaosheng Hu, Shanzhong Zhang.

**Data curation:** Xiaosheng Hu, Jinquan Li.

**Formal analysis:** Xiaosheng Hu.

**Investigation:** Xiaosheng Hu.

**Methodology:** Xiaosheng Hu, Shanzhong Zhang.

**Project administration:** Xiaosheng Hu, Shanzhong Zhang, Jinquan Li.

**Resources:** Xiaosheng Hu.

**Software:** Xiaosheng Hu.

**Supervision:** Shanzhong Zhang.

**Validation:** Xiaosheng Hu.

**Visualization:** Xiaosheng Hu.

**Writing – original draft:** Xiaosheng Hu.

**Writing – review & editing:** Shanzhong Zhang.
